# SPR Biosensor Based on Polymer Multi-Mode Optical Waveguide and Nanoparticle Signal Enhancement

**DOI:** 10.3390/s20102889

**Published:** 2020-05-20

**Authors:** Johanna-Gabriela Walter, Alina Eilers, Lourdes Shanika Malindi Alwis, Bernhard Wilhelm Roth, Kort Bremer

**Affiliations:** 1 Institute of Technical Chemistry, Leibniz University of Hannover, 30167 Hannover, Germany; walter@iftc.uni-hannover.de (J.-G.W.); eilers@iftc.uni-hannover.de (A.E.); 2 School of Engineering and the Built Environment, Edinburgh Napier University, Edinburgh EH10 5DT, UK; L.Alwis@napier.ac.uk; 3 Cluster of Excellence PhoenixD (Photonics, Optics, and Engineering—Innovation Across Disciplines), 30167 Hannover, Germany; bernhard.roth@hot.uni-hannover.de; 4 Hannover Centre for Optical Technologies, Leibniz University of Hannover, 30167 Hannover, Germany

**Keywords:** surface plasmon resonance (SPR), sensor, planar-optical, multi-mode, waveguide, biosensor, aptamer, gold-nanoparticle, lab-on-a-chip

## Abstract

We present a surface plasmon resonance (SPR) biosensor that is based on a planar-optical multi-mode (MM) polymer waveguide structure applied for the detection of biomolecules in the lower nano-molar (nM) range. The basic sensor shows a sensitivity of 608.6 nm/RIU when exposed to refractive index changes with a measurement resolution of 4.3 × 10^−3^ RIU. By combining the SPR sensor with an aptamer-functionalized, gold-nanoparticle (AuNP)-enhanced sandwich assay, the detection of C-reactive protein (CRP) in a buffer solution was achieved with a response of 0.118 nm/nM. Due to the multi-mode polymer waveguide structure and the simple concept, the reported biosensor is well suited for low-cost disposable lab-on-a-chip applications and can be used with rather simple and economic devices. In particular, the sensor offers the potential for fast and multiplexed detection of several biomarkers on a single integrated platform.

## 1. Introduction

The detection of biomarkers in body fluids plays a vital role in early diagnosis and treatment of diseases. However, the potential of biomarkers for the said purpose is not explored to its full capacity due to limitations in the current sensing technologies [1]. This results from the fact that biomarkers are often present at very low concentrations in combination with other proteins, making their identification a strenuous task. In addition, the detection of a particular biomarker at very low concentrations is not only challenging, but also time-consuming [2]. Current methods of detection include enzyme-linked immunosorbent assays (ELISA), surface plasmon resonance (SPR) spectroscopy, surface enhanced Raman spectroscopy and fluorescence-based detection [2,3,4,5,6,7,8]. Amongst these, SPR presents the most advanced label-free and real-time biomarker detection capability [9,10,11,12,13]. The ability of SPR to monitor the interaction between a molecule immobilized on the surface of the sensor and the molecular partner in a solution has rendered it a powerful tool for biomolecular interaction analysis [14].

The classic Kretschmann configuration, a high refractive index prism whose one surface is coated with a thin metallic layer, is commonly applied for exciting SPR, where the incident light intensity that satisfies the plasmonic condition (resulting in the excitation of surface plasmons of the metal), changes in response to variations in the surrounding refractive index. However, in this configuration the required SPR spectrometer is relatively bulky, costly and, thus, restricted to applications in laboratory environments [15]. Since the miniaturization of the detection scheme, in the interest of portability, would be complex, its use in remote sensing or lab-on-a-chip applications would be limited. In comparison, optical fiber-based SPR sensors [16] have the advantages of being small size, offering flexibility in integration and requiring much lower amounts of the analyte sample. A comprehensive amount of work and analysis on optical fiber-based sensors utilizing SPR for applications from chemical- to bio-sensing and its progress within the last decade can be found in the literature [17,18,19]. With the biomedical sector drawing more and more towards lab-on-a-chip schemes, the optical fiber-based biosensor technology is currently seeing a trend towards integration into “micro-chips” that has the capability of multi-parameter sensing in the future [18,20].

Such integrated planar optical waveguide-based biosensors entail label-free and non-destructive detection, higher sensitivity and a lower detection limit, cost efficiency and simple production, as well as multiplexing and miniaturization capabilities which lead to low reagent consumption, short analysis time and open prospects for point-of-care applications [20]. In particular, SPR sensors based on integrated optical polymer waveguides [21] offer all the aforementioned advantages, the most prominent feature being the multiplexed detection capability of several analytes [22,23]. For example, immobilization of biorecognition elements, i.e., on the gold surface of the SPR sensor, can be performed using microfluidic channels where the whole functionalization process or its final steps involving delivery of biorecognition elements to a specific sensing channel is performed with the on-chip microfluidics [14]. Moreover, several SPR sensors can be operated in parallel on a relatively small spatial area and potentially several biomarkers can be detected per sample simultaneously. For instance, single-mode (SM) waveguide based SPR sensors have been reported that are fabricated using a polymer imprinting process [22,24] or by using the spin coating and photolithography process [23].

On the other hand, multi-mode (MM) waveguide structures have the advantage that, due to the relatively large cross-section of the waveguide core, light coupling in and out of the structure is less critical compared to the single-mode case. Thus, this kind of photonic device is especially suited for mass-market products such as disposable lab-on-a-chip devices that can be interrogated by using a low-cost light source and spectrometer, such as the flash light and camera electronics that are readily available in smartphones [25]. Indeed, this forms the basis of the novelty presented herein, i.e., the utilization of the already in-built electronics of a commonly available device that is used by almost the entire world and the evaluation of its potential for biosensing. This approach cuts down the potential cost of interrogation, i.e., as the source/detector is already in-built and equipped with appropriate software, making it a viable option for cost efficient mass production. In addition, the proposed design could transfer conventional SPR sensing, which is currently restricted to laboratory settings, into the global consumer world, through the careful combination of an integrated polymer waveguide, SPR sensors and the in-built electronics of a smartphone.

However, when optical MM waveguides are utilized for SPR sensor applications each mode of the waveguide structure couples at a slightly different wavelength with the surface plasmons (the corresponding propagation constants match at different wavelengths), and thus, the resulting SPR spectrum becomes relatively broad. This, in turn, limits the resolution to refractive index changes of the surrounding media since the exact minimum position of the resonance is more difficult to track due to the relatively broad resonance and modal noise. Therefore, biomarker detection in the nanomolar (nM) concentration range is usually difficult to achieve with this kind of technology, particularly bearing in mind that the main performance indicators for plasmonic biochemical sensors are not only their sensitivity, but also their accuracy, repeatability and limits of detection (LOD) [3,26,27].

The proposed design addresses these important issues through the novel combination of planar-optical MM polymer waveguide SPR sensors and aptamer-assisted gold nanoparticle (AuNPs)-enhanced sandwich assays, which ultimately enables the detection of biomolecules down to nM concentrations. The combination of SPR spectroscopy and AuNP enhanced sandwich assays was proven to be a very powerful tool to achieve high sensitivities [28,29,30], even down to femtomolar (fM) concentrations [31]. However, to the best of our knowledge, the combination of planar-optical MM polymer waveguide SPR sensors and aptamer-assisted gold nanoparticle (AuNPs)-enhanced sandwich assays has not been investigated to date, thus providing an opportunity for the novel approach suggested. We demonstrate the capability of this combination as a new technique for biomarker detection.

To demonstrate the functionality of the sensor, C-reactive protein (CRP) was chosen as a model analyte, which was detected in buffer solutions using an aptamer-based and gold nanoparticle (AuNP)-enhanced sandwich assay. CRP is an acute phase response protein that represents an inflammation marker and can be used for monitoring of exacerbations in chronic inflammatory conditions [32]. Clinically, CRP detection is used to differentiate the infection caused by bacteria from virus [33]. High levels of CRP are observed after trauma, tissue necrosis, infection, surgery and myocardial infarction and are associated with an increased risk of cardiovascular diseases, especially coronary heart disease risk, which is a leading cause of death in adults [34]. Due to its importance in the diagnosis of diseases, the detection of CRP in a lab-on-a-chip scheme would be highly attractive to medical sectors. Even though to date, several methods have been developed for CRP detection [35,36], including SPR-based sensing [37], the optical waveguide-based detection schemes designed for this purpose are mostly focused on the sensor (or indeed the sensing region) being an optical fiber as opposed to being incorporated in a waveguide structure [33,34].

In light of the discussion presented above, the goal of this study is to demonstrate a planar SPR sensor using integrated polymer MM waveguides that can be interrogated using a smartphone in the future. Consequently, the strengths of the herewith proposed scheme are its adaptability and thus its high potential in utilizing a single “chip” for multi-biomarker sensing and the higher tuning of the detection scheme, as well as potentially low-cost interrogation by using the source/detection electronics that are readily available in a portable device, i.e., even in the simplest of smartphones currently available [25]. Together with initial laboratory results and analysis, the communication herein presents the design incorporating the MM waveguide SPR sensor in tandem with an aptamer-assisted AuNP-enhanced assay with CRP as a model biomarker.

## 2. Sensor Design and Fabrication

**Figure 1:**
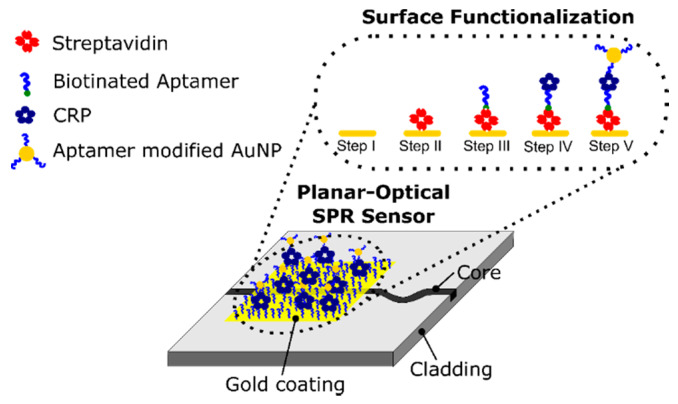
Schematic of the developed polymer based multi-mode (MM) planar-optical waveguide surface plasmon resonance (SPR) sensor. By using a gold-nanoparticle (AuNP)-enhanced aptamer-based sandwich assay, the shift of the SPR wavelength due to the binding of the target molecule (in this case C-reactive protein (CRP)) is increased.

For the specific detection of the biomarker, aptamers were employed for sensor functionalization. Suitable capture aptamers were immobilized on the sensor surface and detection aptamers were conjugated to AuNPs resulting in an AuNP-enhanced sandwich system. Since CRP is a pentameric protein consisting of five identical subunits, the same aptamer could be exploited for capture and detection in our approach. A schematic of the proposed sensor configuration is shown in Figure 1.

The planar-optical MM polymer waveguide structure is fabricated using a combination of hot embossing and doctor blading (see Section 3.1; details can also be found in [38,39]). For detection of CRP, a specific surface functionalization is applied (explained in Section 3.2). Due to the binding of the AuNPs on the target molecule, the shift of the SPR wavelength is significantly enhanced and hence detection of the molecule at low concentrations is ultimately feasible despite the modal noise exhibited by the MM waveguide structure. This would not be possible with the setup otherwise.

## 3. Materials and Methods

### 3.1. Fabrication of Planar-Optical SPR Waveguide Sensors

The fabrication of the planar-optical SPR waveguide sensor consists of two steps. In the first step, the planar-optical waveguide structure is manufactured. This step is followed by the coating of the waveguide surface with a thin gold film. The fabrication of the waveguide is based on the technique developed by Rezem et al. and is described in detail elsewhere [38,39]. Here, we briefly summarize the main points. First, a silicon wafer stamp containing a negative copy of the structure to be realized is employed to replicate the waveguide cladding structure into a 375 µm thick PMMA sheet (Plexiglas XT 99524, Röhm GmbH, Darmstadt, Germany) using hot embossing. After the embossing process, the so-called doctor blading method is applied to fill the optical waveguide cladding structure with liquid optical epoxy material, which has a higher refractive index compared to PMMA, in order to form the optical waveguide structure. The waveguide core is solidified through illumination with a UV light source (UV Transilluminator MUV21, Major Science, Saratoga, NY, USA). In our case, we employed the UV curable epoxy NOA 63 with a refractive index of 1.56 as waveguide core material. Optical waveguide structures fabricated with this technique and the above materials exhibit an optical attenuation as low as 0.037 dB/mm which is obtained by characterization through the cut-back method [38].

In the second stage of the fabrication process, the surface of the planar-optical waveguide structure was coated with a 40 nm thick gold layer. In order to achieve a strong bonding between the applied polymers and the thin gold layer, a thin layer of titanium (2 nm) was applied as an adhesive layer. The total length of the planar-optical waveguide structure was 40 mm and the dimension (cross-section) of the waveguide itself was 25 × 25 µm^2^. As shown in Figure 1, a bending region was introduced to the waveguide (with a bending radius of 5 mm) in order to enhance mode coupling and consequently obtain a more uniform mode energy distribution. After the fabrication process of the planar-optical SPR waveguide sensor structure was complete, a microfluidic chip (Ibidi sticky-Slide VI 0.4, Ibidi, Munich, Germany) containing four microfluidic channels was attached and glued on top of the gold surface. Each microfluidic channel has a length of 17 mm, a width of 3.8 mm and a height of 400 µm. The microfluidic channels were aligned on the gold surface so that the direction of the fluid flow containing the target molecules to be detected is perpendicular to the planar-optical waveguide direction. Thus, the resulting length of each SPR sensor per channel is 3.8 mm. The fluid inlet and outlet of the microfluidic chip are standardized female Luer adapters.

### 3.2. Surface Functionalization for the Detection of CRP

The gold surface was coated with streptavidin in the first step of the developed functionalization procedure. For this purpose, streptavidin (Roth GmbH, Germany) was diluted in phosphate buffered saline, pH 7.4 (PBS) at a concentration of 2.5 mg/mL, and the solution was incubated on the sensor surface for 1 h at room temperature and additionally for 16 h at 4 °C. Consequently, the sensor was washed thoroughly with PBS and rinsed 2 times with ddH_2_O before it was dried with compressed nitrogen. Streptavidin modified sensors were stored at 4 °C in a dry environment prior to use.

Then, Anti-CRP aptamer Apt1 with the sequence GGCAGGAAGACAAACACGATGGGGGGGTATGATTTGATGTGGTTGTTGCATGA-TCGTGGTTGTGGTGCTGT [40] and 5′termial biotin modification (IDT) was used for further modification of the sensor. In order to do so, the biotinylated Apt1 was diluted to 1 µM in PBS and incubated on the sensor surface for 1 h at room temperature to bind to the sensor via streptavidin-biotin interaction. Afterwards, unbound aptamers were removed by rinsing the sensor with PBS two times and one additional washing step using binding buffer (BB) composed of 10 mM Tris, 50 mM NaCl, 2 mM CaCl_2_, 10 mM MgCl_2_, 5 mM KCl, pH 7.5.

### 3.3. Synthesis and Modification of AuNPs

AuNPs were prepared via kinetically controlled seeded growth synthesis as described by Bastús et al. [41] with ten consecutive rounds of additions of HAuCl_4_ and sodium citrate. This resulted in a colloidal gold solution with a light absorption maximum at 522 nm. The size of the produced AuNPs was determined to be 17 nm via transmission electron microscopy (TEM) using a field-emission instrument of the type JEOL JEM-2100F-UHR. The AuNPs were diluted to obtain a light absorption at the absorption maximum (Amax) of 0.7, and streptavidin was added to a final concentration of 50 µg/mL in 10 mM sodiumphosphate pH 7.4. To avoid agglomeration of the AuNPs during adsorption of streptavidin, the AuNPs were stirred during the addition of streptavidin and for an additional hour at room temperature. The resulting mixture was stored at 4 °C for 16 h. Consequently, residual streptavidin was removed via centrifugation and the conjugates were washed with ddH_2_O. Biotinylated Apt1 was added to the streptavidin-modified AuNPs (Amax = 3.5) at a final concentration of 10 µM to be bound at the NP surface. After overnight incubation, free residual aptamer was removed using centrifugation and washing in ddH_2_O, and a stock solution of the aptamer-modified AuNPs (Amax = 2) was prepared.

### 3.4. Experimental Setup

**Figure 2:**
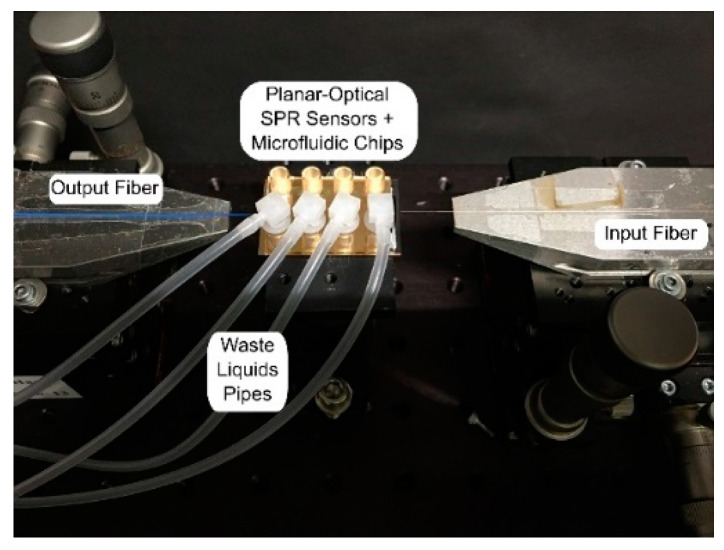
Picture of the experimental setup showing the planar-optical waveguide SPR sensor including the microfluidic chip (middle) as well as the two optical glass fibers for light coupling into (right) and out (left) of the structure.

The SPR sensor spectrum was captured in transmission mode using a cost-effective white light LED (Thorlabs MCWHF2) and an optical spectrometer (Avantes AvaSpec-3648, Avantes, Apeldoorn, Netherlands). The light coupling into and out of the planar-optical SPR waveguide sensor was achieved using optical glass fibers. For the light coupling into the waveguide structure, tapered graded-index MM optical glass fibers (OM4) with a spot diameter of 25 µm (Thorlabs LFM100. Thorlabs, Newton, NJ, USA) were applied. In order to collect the light that is coupled out of the planar-optical waveguide structure, a step-index MM fiber with a numerical aperture of NA = 0.5 was used (Thorlabs FP200URT. Thorlabs, Newton, NJ, USA). The alignment of the optical fibers relative to the planar-optical SPR waveguide sensor was realized using a linear stage (Thorlabs RBL13D/M. Thorlabs, Newton, NJ, USA) for each fiber. The experimental setup used is presented in Figure 2, which shows the planar-optical waveguide SPR sensor with microfluidic chip as well as the light coupling in and out of the structure by the optical glass fibers.

For each measurement, the sensor spectra were recorded using the spectrometer software Avantes avasoft8, and the captured spectra was analyzed using Matlab. Following this, the signal noise of the sensor spectra was reduced using a smooth-average filter operation (N = 30), and the position of the SPR wavelength was calculated using the “center of mass” method [42], i.e., the wavelength of the measured intensity minimum (maximum light coupling between waveguide and surface plasmon) in the transmission spectrum was tracked.

### 3.5. CRP Sensing

The aptamer-modified sensor was exposed to different concentrations of CRP purified from human plasma (BioRad) in the BB. For each measurement, 50 µL of the solution was incubated on the sensor for 30 min. Non-bound CRP was removed by rinsing the sensor with 600 µL of BB and 50% BB respectively. Afterwards, aptamer-conjugated AuNPs were diluted to A_525_ = 1 in BB, and the sensor was exposed to 50 µL of this solution for 10 min. Finally, the sensor was washed with 600 µL of 50% BB and H_2_O before the readout was performed in H_2_O. To investigate unspecific binding, the sensor was exposed to 10% CRP-free human serum (HyTest Ltd., Turku, Finland) in BB for 30 min before AuNP-based signal enhancement was performed as described above.

## 4. Results

### 4.1. Response of the Polymer Based Planar MM Optical SPR Sensor to RI Changes

**Figure 3:**
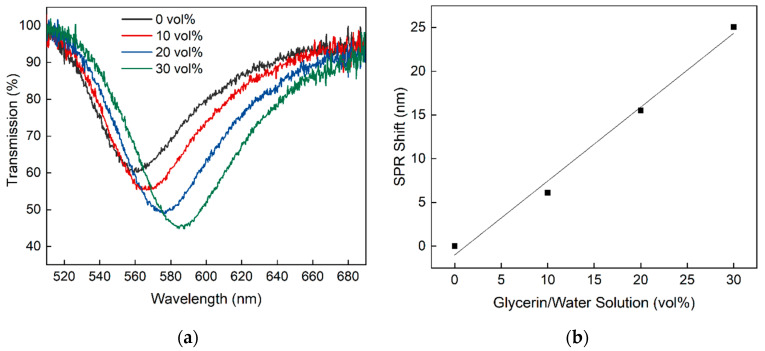
Response of the planar-optical MM SPR waveguide sensor to different glycerin/water solutions. (**a**) The resulting transmission spectrum for different glycerin/water solutions and (**b**) the corresponding SPR wavelength shift for each transmission spectrum.

For the initial characterization of the sensitivity of the planar-optical waveguide SPR sensor, different refractive index (RI) solutions of glycerin and water with volume percent (vol%) of glycerin (0%, 10%, 20% and 30%) were applied as described in [25]. The fabricated planar-optical SPR sensor was tested with different glycerin/water solutions and the obtained SPR spectra as well as the shift of the SPR wavelength to the different glycerin/water solutions were determined. This is depicted in Figure 3. The measured spectra and the center of the SPR wavelength were obtained using the algorithm explained in Section 3.4.

From Figure 3 it follows that the SPR wavelength shifts towards higher wavelengths with increasing glycerin concentrations. This behavior agrees well with other optical waveguide-based SPR sensors reported in the literature. Furthermore, a linear behavior of 0.845 nm/vol% (R^2^ = 0.99) could be measured, which is equal to 608.6 nm/RIU when expressed in refractive index units (RIU) [25].

### 4.2. Signal Noise of Polymer Based MM Planar Optical SPR Sensor

The signal noise of the polymer-based MM optical waveguide SPR sensor was evaluated with the experimental setup described in Section 3.4 and by applying water as a refractive index medium (n = 1.33) as well as taking nine measurements. When conducting the signal noise tests, a standard deviation of 0.87 nm was obtained for the measured SPR wavelength (calculation of the minimum position in the measured SPR transmission spectrum was performed using the algorithm described in Section 3.4). When applying the equation for determining the LOD according to Hastings [26], a LOD of 4.3 × 10^−3^ RIU for the detection of RI was obtained. The relatively high LOD, i.e., compared to Suzuki et al. [43] who reported a LOD of 2.3 × 10^−5^ RIU, can be explained by modal noise of the MM waveguide structure. As described in Section 3.4, optical glass fibers were applied for the purpose of light coupling, i.e., light into and out of, to the planar-optical waveguide. Since the position of the fibers relative to the core of the planar-optical waveguide is sensitive to external perturbations such as slight temperature variations or vibrations, minor spatial fiber-to-waveguide position variations cause different modal excitations in the waveguide and thus variations in the detected SPR wavelength (minimum position of the measured intensity transmission spectrum).

### 4.3. Detection of CRP Concentration

**Figure 4:**
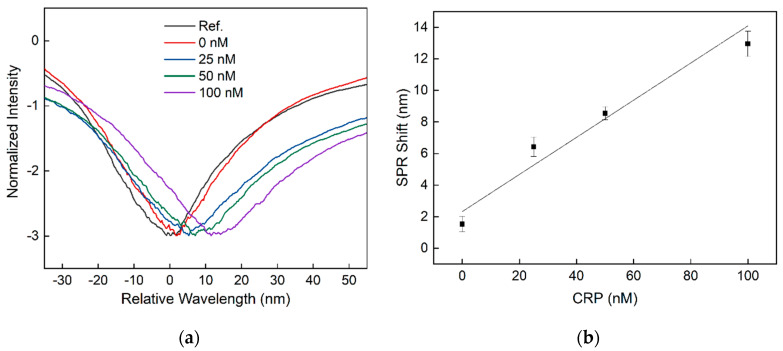
Transmission spectra (**a**) and SPR wavelength shift (**b**) for different CRP concentrations in combination with the applied AuNP sandwich assay.

In order to demonstrate the capability of the developed sensor concept to detect biomarkers with high sensitivity, the aptamer-assisted AuNP-enhanced sandwich assay was applied and evaluated using the acute phase protein CRP and a DNA aptamer directed against CRP. For this purpose, the gold surface of the planar-optical SPR sensor was modified according to the protocols described in Section 3.2 (steps II and III in Figure 1). The sensor response was determined again using the experimental setup and the algorithm explained in Section 3.4. The SPR wavelength was measured with water (n = 1.33) as a surrounding buffer medium before and after the addition of CRP and aptamer-modified AuNPs for signal enhancement, see Section 3.5 (steps IV and V in Figure 1). When only CRP has bonded to the sensor surface, no shift of the SPR wavelength could be detected and consequently the SPR sensor structure itself was not capable of detecting biomarkers in the nM range. However, by applying AuNPs additionally, a clear shift in the SPR wavelength was observed. The obtained SPR spectra for different CRP concentrations and for water (without incubation with CRP and AuNPs as reference) are illustrated in Figure 4a.

According to the results shown in Figure 4a, the SPR wavelength shifts towards higher wavelengths with increasing CRP concentration and, thus, for higher amounts of bound AuNPs. The shift of the SPR wavelength towards higher wavelengths with increasing amounts of AuNPs above the sensor surface is consistent with other SPR sensor systems based on AuNP sandwich assays reported in the literature [44] due to electromagnetic field coupling between SPR and localized SPR (LSPR) of the AuNPs [45]. With the enhanced sensor configuration, a sensitivity of 0.118 nm/nM (linear approximation with R^2^ = 0.95) for the detection of CRP in buffer solution could be obtained (Figure 4b). As depicted in Figure 4b, for each CRP concentration, a new sensor channel was applied, and for the determination of the SPR wavelength shift, a mean value was calculated over nine measurements. In addition, in order to determine the LOD of the CRP detection, the standard deviations were calculated for all measurements and values of 0.49 nm (0 nM), 0.62 nm (25 nM), 0.42 nm (50 nM) as well as 0.81 nm (100 nM) were obtained. By taking the standard deviation for 0 nM CRP and the obtained sensitivity, a LOD for the detection of CRP of 12.46 nM was calculated. For samples with 0 nM CRP and spiked with 10% CRP-free human serum, a non-specific binding induced SPR wavelength shift of 6.13 nm was measured.

## 5. Discussion

Our evaluation verifies that the proposed planar-optical SPR sensor design has a linear sensitivity of 608.6 nm/RIU to applied RI changes of the surrounding and a LOD of 4.3 × 10^−3^ RIU in its basic configuration. The relatively moderate measurement resolution to applied RI changes achieved can mainly be explained by the modal noise of the MM optical waveguide structure. Since optical glass fibers were applied for the purpose of coupling light in and out of the waveguide, their positions relative to the planar-optical waveguide structure are sensitive to external perturbations, i.e., temperature variations or vibrations. Therefore, spatial variations of the fiber-to-waveguide position cause different mode excitations of the planar-optical waveguide structure, which results in variations in the detected SPR wavelength (minimum position of the measured intensity transmission spectrum). However, despite this fact, the detection capability of the target biomarker CRP in the lower nM range could be demonstrated by extending the sensor configuration with a AuNP-enhanced aptamer-based sandwich assay, and thus, CRP concentrations lower than 25 nM (the lowest concentration used in this study) can be reliably detected with a LOD of 12.46 nM. The normal CRP concentration in human serum is 40 nM, and CRP concentration increases above 666 nM (840 mg/L) in the case of inflammation and infection [46]. The sensitivity of the developed sensor is thus well-suited for the detection of CRP within the physiologically relevant concentration range and, in particular, to distinguish between normal and increased CRP levels.

## 6. Conclusions

A SPR biosensor based on a planar-optical MM polymer waveguide for the detection of biomolecules in the lower nM range was successfully developed. Through the novel combination of the planar-optical SPR sensor with an aptamer-based and AuNP-enhanced sandwich assay, detection of a model biomarker, i.e., C-reactive protein (CRP), was demonstrated. While CRP was only used as a model analyte and neither the surface functionalization nor the assay procedure were optimized, a sensitivity of 0.118 nm/nM and a limit of detection of 12.46 nM were achieved, which would not have been possible with the basic sensor concept, i.e., without the NP-assisted signal enhancement concept developed in this work. Furthermore, depending on the functionalization of the gold surface of the SPR sensor, the detection of various other biomarkers appears feasible in the next step. Therefore, by multiplexing several SPR sensors in parallel with different surface functionalization, multiple biomarker detection is possible within a relatively small spatial area and requiring only a small sample volume. Furthermore, the MM optical waveguide structure was fabricated using hot embossing and doctor blading, and thus, the fabrication of a whole planar-optical SPR biosensor system can be transferred to a cost-efficient and potentially reel-to-reel fabrication process with high throughput. Moreover, the design demonstrated the potential for implementing cost-efficient coupling of light with the all-optical sensor chip, which is important for the reduction of the overall interrogation cost of the sensing scheme. In the present study, we have used a white light LED and a spectrometer for interrogation and readout to demonstrate the functionality of the developed sensor system. Since no sophisticated optical instruments are needed, the sensor can also be used in combination with a mobile device, where the LED and the camera of a smartphone, for instance, can be employed for interrogation, as had been demonstrated before for a fiber optic SPR sensor [25]. This will reduce costs of the needed devices dramatically in comparison to conventional SPR spectrometers and will allow the application of SPR outside laboratory environment and within low-resource settings.

In summary, the novel biosensor incorporating MM polymer waveguides demonstrates strengths in its adaptability for multi-biomarker sensing on a single microfluidic chip, cost effectiveness, i.e., not only in the fabrication but also in its requirement of minimal sample volumes, and its higher tuning of the detection scheme. It can be concluded that it is well-suited and, most importantly, has the potential for low-cost disposable lab-on-a-chip applications which might be of particular interest in low-resource settings. Current research focuses on the sensor system to be incorporated into a smartphone and the optimization of the assay for the detection of biomarkers in complex samples as well as optimization of the sensitivity by tailoring the interaction between the SPR and LSPR of the AuNPs.
